# Impact of lunch with carbohydrates differing in glycemic index on children's cognitive functioning in the late postprandial phase: a randomized crossover study

**DOI:** 10.1007/s00394-021-02766-y

**Published:** 2021-12-13

**Authors:** Alina Drozdowska, Kathrin Sinningen, Michael Falkenstein, Henrik Rudolf, Lars Libuda, Anette E. Buyken, Thomas Lücke, Mathilde Kersting

**Affiliations:** 1 Research Department of Child Nutrition, University Hospital of Pediatrics and Adolescent Medicine, St. Josef-Hospital, Ruhr-University Bochum, Alexandrinenstraße 5, 44791 Bochum, Germany; 2 ALA Institute, Bochum, Germany; 3 Department of Medical Informatics, Biometry and Epidemiology, Ruhr-University Bochum, Bochum, Germany; 4 Paderborn University, Faculty of Natural Sciences, Institute of Nutrition, Consumption and Health, Nutrition Sciences, 33098 Paderborn, Germany; 5 Paderborn University, Faculty of Natural Sciences, Institute of Nutrition, Consumption and Health, Public Health Nutrition, 33098 Paderborn, Germany

**Keywords:** Dietary glycemic index, Lunch, Cognition, Schoolchildren

## Abstract

**Purpose:**

Studies about effects of lunch dietary Glycemic Index (GI) on cognition of schoolchildren are scarce. Our previous CogniDo GI study found no changes of cognition in the early postprandial phase after consumption of two rice types with medium vs. high dietary GI for lunch (i.e., 45 min after starting lunch). This study investigated whether the dietary GI of lunch has an impact on cognition of schoolchildren in the late postprandial phase, 90 min after lunch.

**Methods:**

A randomized, 2 × 2 crossover intervention study was conducted at a comprehensive school with 5th and 6th grade students. Participants (*n* = 212) were randomly assigned to either sequence 1 or 2. In the first period, participants of sequence 1 received a dish with high GI rice (GI: 79), those of sequence 2 with medium GI rice (GI: 64)—in the second period, 1 week later, vice versa. Computer-based cognitive testing was performed 90 min after lunch examining tonic alertness, visual search and task switching, and working memory. Treatment effects and treatment effects adjusted for estimated lunch glycemic load (GL) were analyzed using a linear mixed model.

**Results:**

The selected cognitive parameters were not affected by the GI of lunch 90 min after lunch, neither after intention-to-treat nor in the per-protocol analysis. Adjustment for GL also did not change results.

**Conclusion:**

The present study revealed no notable differences after the consumption of two rice types with medium vs. high dietary GI for lunch in children’s cognitive function in the late postprandial phase, 90 min after lunch.

**Clinical trial registration:**

German Clinical Trials Register (DRKS00013597); date of registration: 16/04/2018, retrospectively registered.

**Supplementary Information:**

The online version contains supplementary material available at 10.1007/s00394-021-02766-y.

## Introduction

Remaining concentrated and attentive throughout a school day can be challenging for schoolchildren. Nutrition might help to sustain or even promote their cognitive performance and indeed, several dietary factors have been proposed to be beneficial. For instance, fatty fish consumption and omega-3 fatty acid intake have been associated with improved memory and faster processing speeds [1, 2]; a sufficient water supply was shown to have favorable effects on cognitive performance [3], and some studies indicate that breakfast composition with regard to glycemic carbohydrates might influence cognition in children throughout the morning [4, 5], as well as in adults suffering from impaired glucoregulation [6]. Especially different carbohydrates were studied with regard to their Glycemic Index (GI). The GI ranks available glycemic carbohydrates provided by carbohydrate-rich foods by their effects on postprandial blood glucose concentrations. The Glycemic Load (GL) reflects the glycemic response to the carbohydrates in a given portion or meal consumed (product of GI x carbohydrates in that portion/meal). The reason why carbohydrate-rich foods are thought to influence cognition depending on their GI is that the brain is sensitive to changes in blood glucose concentrations [7]. Especially foods with lower dietary GI are presumed to act favorably, probably because of more sustained, longer-lasting rises in blood glucose concentrations compared to high GI foods, yielding rapid increases of blood glucose concentrations [8]. For instance, Ingwersen et al*.* observed a significant decline in attention in children 2 h after consumption of a breakfast with high GI foods, while a breakfast with low GI foods showed a less decline in secondary memory [4]. Similarly, a breakfast with low GI foods was accompanied by improved short-term memory and better verbal auditory attention [9]. The study from Micha et al*.* revealed that a breakfast with low GI foods led to better verbal fluency in children, while breakfast with high GI foods had a positive impact by improving the speed of information processing 90 min after breakfast [10]. Correspondingly, consumption of a high GI cereal was associated with better memory 90 min after breakfast in another study [11]. Thus, the existing literature on GI of breakfast and cognition seem to vary depending on the time of testing and the parameters examined.

While the impact of estimated breakfast GI has been studied extensively, studies on estimated dietary GI of lunch are scarce. In our previous study, we were the first to examine whether the dietary GI of foods consumed at lunch influences children’s cognition in a similar way as breakfast [12]. No differences were detected 45 min after consumption of a carbohydrate-rich dish with either medium or high dietary GI rice. In addition, we have shown in the past that, contrary to findings in adults, children's cognition was not negatively influenced by lunch [13]. To the best of our knowledge, no studies have yet examined the effects of lunch GI on schoolchildren's cognition 90 min after lunch, comparable to studies examining the effects of different dietary GI of breakfasts [10, 11].

Based on the conflicting results and insufficient evidence of other studies regarding improvement in memory and selective attention in the late postprandial period (75, 90 min or later) after the breakfast [11, 14–17], we hypothesized that children's cognitive performance after lunch varies depending on the GI of the meal as well. Thus, the aim of this CogniDO GI II (Cognition Intervention Study Dortmund Glycemic Index Part II) study was to examine whether lunches differing in estimated dietary GI influence the cognitive performance of schoolchildren 90 min after beginning of lunch, i.e., doubling the interval between lunch and cognitive testing compared to our previous study [12].

## Methods

### Study design and recruitment

The CogniDO GI II study was designed as a randomized, single blind 2 × 2 crossover intervention study in accordance with previous CogniDo studies that investigated the impact of lunch per se on cognitive performance as well as the impact of lunch with different dietary GI after 45 min [12, 13, 18, 19]. Recruitment of participants from all 5th and 6th grade classes (13 classes; participants age approx. 10–12 years) was undertaken from September 2017 until January 2018 at the ‘Comprehensive School Berger Feld’ in Gelsenkirchen, Germany. Participants with a metabolic disease or a diagnosed learning disorder were excluded.

Within our 2 × 2-crossover study, participants were assigned within their class (13 classes with approximately 26–30 students each) upon receiving the consent letter into one of two sequences using simple randomization stratified by sex. For allocation, a computer-generated list of random numbers was used. Thereupon, participants from sequence 1 received lunch with high GI rice (hGI) on the first study day and medium GI rice (mGI) on the second study day, after a 7 day washout period (sequence h-mGI); participants of sequence 2 were treated vice versa (sequence m-hGI). The field period lasted from October 2017 to January 2018 excluding school holidays.

In accordance with the previous CogniDo GI I study [12], the GI values of both rice types were analyzed by the Sydney University Glycemic Index Research Service (SUGiRS), a certified laboratory for GI testing (ISO 26642:2010). Briefly, a group of 11 healthy volunteers (aged 18–65 years) received either glucose or the two rice samples containing 50 g of digestible carbohydrates on three different days after overnight fasting. Capillary blood samples were obtained from each subject constructing a 2-h plasma glucose response curve. Finally, GI values for each rice sample relative to the reference (glucose) were calculated using the incremental area under the 2-h plasma glucose response curve (iAUC).

**Fig. 1:**
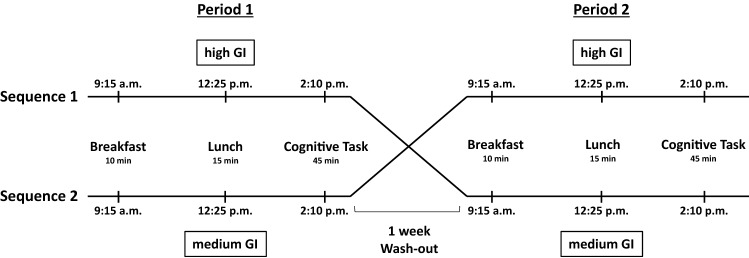
Schedule of the crossover study. Each test day started at 9:15 a.m. with a standardized breakfast. In period 1, participants from sequence 1 received high GI rice for lunch at 12:25 p.m., participants from sequence 2 medium GI rice. At 2:10 p.m. cognitive testing started. In period 2, after 1 week wash-out, participants from sequence 1 received medium GI rice, participants from sequence 2 high GI rice

A standardized breakfast (bread from wholemeal flour, margarine, poultry salami or Gouda cheese and carrot sticks) was offered ad libitum at 9:15 am to all subjects at both test days to ensure comparable preconditions. Beforehand, parents were instructed to send their children to school without breakfast on the day of the test. At the beginning of lunch break (12:25 pm), both sequence-groups received a carbohydrate-rich dish of rice and ground beef sauce (ad libitum). Meal compositions were identical except for the rice type. Participants of sequence 1 received rice with hGI (GI = 79; Jasmine Rice, Müllers Mühle) and sequence 2 received rice with mGI (GI = 64; Basmati Rice, Oryza Himalaya). On the second test day, 1 week later, rice types were switched between the sequence-groups (Fig. 1). Time allocated to eat lunch was 15 min. The amount of consumed lunch was documented by the study staff. We excluded all participants who did not eat lunch or rice at all. The plates were weighed before and after the meal. As a prescribed amount of food might have negatively affected children’s well-being and cognitive performance, the portion size of the meal was not standardized and participants were allowed to eat ad libitum. Therefore, the estimated meal glycemic load (GL) of the consumed rice portion was obtained by multiplying the amount of rice-carbohydrates consumed by the GI of the respective rice (GL = GI x carbohydrate content (g) per portion/100). Carbohydrates provided by the sauce were ignored.

Between breakfast and cognitive assessment, participants were instructed not to consume any other food or beverages except the study lunch, water or unsweetened fruit or herbal tea. Participants were questioned about their eating and drinking behavior on the study day at the end of the cognitive tests.

The study was conducted according to the Declaration of Helsinki and was approved by the Ethical committee of the Medical Faculty, Ruhr University Bochum, Germany (approval number 17-6123). Written informed consent was obtained from all parents or legal guardians before the study start, and all children gave assents to participate.

### Cognitive assessment

Three computerized cognitive tasks, developed by the ALA Institute in Bochum, Germany, were performed classwise in a quiet room within the school. Before cognitive assessment at 2:10 pm, 90 min after lunch, a pre-test followed by a low activity break was performed (duration approx. 5 min). The pre-test session included the explanation of all tasks and an exercise of approximately 1 min for each task. The children were allowed to ask questions and all testing sessions were performed by the same study staff. Cognitive assessment lasted 45 min, i.e., 90–135 min after lunch. The participants were asked to perform all tasks as quickly as possible without losing accuracy.

#### Task switching

**Fig. 2:**
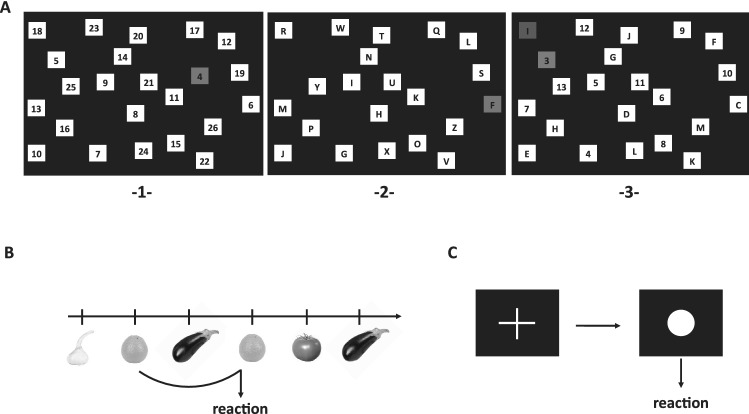
Cognitive tasks. **A** Switch task consisting of 3 sections: **1** numbers had to be clicked in ascending order (non-switch). **2** Letters from A to Z had to be clicked alphabetically (non-switch). **3** Numbers and letters had to be clicked alternately in ascending order (switch, i.e., 1-A–2-B–3-C). **B** 2-back task. Images with fruits and vegetables were displayed on a computer screen. Participants were instructed to press a defined button when an image matched an image 2 trials before. **C** Tonic alertness. When a white circle appeared on a black computer screen participants had to press a button as quickly as possible, the display of a white cross required no reaction

Spatial attention and switching abilities between two different tasks were measured using an alternative version of the Trail Making Task [20] consisting of three trials (Fig. 2A). In trial 1, numbers from 1 to 26 were displayed on a computer screen and had to be clicked in ascending order as quickly and accurately as possible. Trial 2 consisted of letters from A to Z that had to be clicked in alphabetical order. Trial 3 included both numbers (1–13) and letters (A–M) and participants had to alternately click numbers and letters in ascending order (e.g., 1-A–2-B…13-M). Correctly clicked signs turned green and faded out, incorrectly turned red. The task could be continued only if targets were clicked correctly (green feedback). Each trial was limited to 3 min. Reaction time (RT) for non-switch trials and switch trials were measured. Switch costs were defined as the difference in respond time between switch trials and non-switch trial and were calculated as follows: Switch costs = Switch RT [item2–26]—Numbers RT [item 2–26]—[Letters RT (item 2–13)—Numbers RT (item 2–13)]. Negative switch costs indicating inadequate reactions (at least one of the trials was not completed in time) were considered implausible and were excluded from the analysis.

#### 2-back task

Short-term working memory performance and updating was assessed by the visual domain. 106 stimuli (images with fruits and vegetables) were presented consecutively on a computer monitor (Fig. 2B). Children were instructed to press a defined button each time a stimulus matched a stimulus 2 trials before. The test consisted of 21 correct trials. Each stimulus was presented for a maximum 500 ms with an interval of 2100 ms regardless of whether the participant responded within the limited time of 1400 ms or not. There was no positive or negative feedback. RT was calculated only for correctly responded trials. Measures of accuracy were the ratio of false alarms (response to wrong trial) and the ratio of missings (no reaction to correct trial).

#### Tonic alertness

A simple response task was used with two white targets (cross and circle) individually presented on black background in the middle of the screen (Fig. 2C). When a white circle appeared, the participants had to press a button as quickly as possible (maximal RT 1500 ms), the cross required no reaction. The test included 50 targets (circle) with a response stimulus interval of 3300 ms (± 20%). The outcome variables were the mean RT (ms) and the deviation of RT (ms) for speed and the number of omission errors (no reaction after 1500 ms) and the number of commission errors (reaction during the presence of the cross) for accuracy.

### Statistical analyses

All analyses were performed using the statistical software package IBM^®^ SPSS^®^ Statistics for Windows, version 25.0 (IBM Corp., Armonk, N.Y., USA).

Interval-scaled parameters of the cognitive tasks were used as outcome variables (switch task: switch costs, visual search letters, visual search numbers; 2-back task: RT, ratio of missings, ratio of false alarms; alertness: mean RT, Deviation of RT, count of omission errors, count of commission errors). The sums of the two individual values of the outcome variables (cognitive parameters) of period 1 and 2 were compared between both sequence groups using an unpaired *t* test for normally distributed data and the Wilcoxon rank-sum test for non-normally distributed data to examine potential carryover effects of the treatment [21, 22]. Since no carryover effects were observed, results from both days were considered for the treatment effect. Treatment effects were analyzed using a linear mixed model. First, non-normally distributed outcomes were transformed (logarithm, square root, or reciprocal transformation). Period, sequence and GI were treated as fixed effects, subjects as random. With this model period effects (i.e., learning effects) and sequence effects (i.e., randomization effects) were also detected.

Because lunch portion size varied between participants, associations of GI with cognitive parameters were additionally adjusted for GL (fixed effects: GL, period, sequence, GI; random effects: subjects). To overcome Type I error of multiple testing (number of outcome parameter *n* = 10) *p* values were adjusted using Bonferroni–Holm correction. Results are displayed as mean ± standard error of mean (SEM) and 95% confidence interval of mGI and hGI treatment effects and of the treatment difference (mGI–hGI).

Per protocol analysis was performed by excluding participants who did not follow the study protocol.

## Results

### Participants

**Fig. 3:**
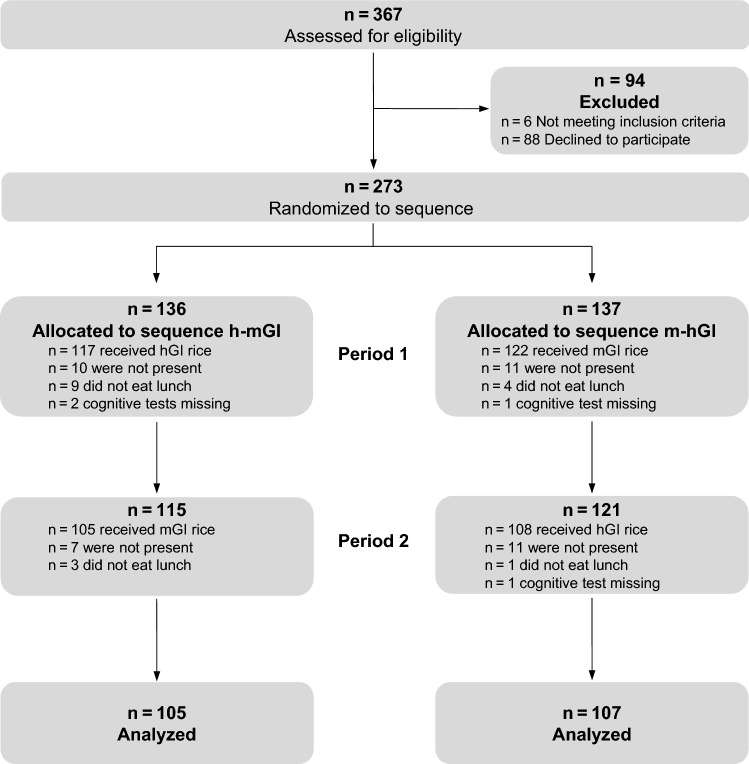
Flow diagram. *hGI* high glycemic index, *mGI* medium glycemic index

Out of 367 eligible children, 279 confirmed their participation and 273 met the inclusion criteria (Fig. 3). Written informed consent was available for 279 children. Six children with learning disabilities were excluded from analyses (*n* = 273). Finally, a modified intention-to-treat analysis was performed with data from 212 children who participated on both experimental days. For per-protocol-analysis, 21 children were excluded who did not follow the instructions for refraining from eating anything besides the study food between lunch and cognitive testing.

Sex distribution and estimated GL, stratified by sequence group (h-mGI and m-hGI) are presented in Table 1. Overall, the average serving size was 284 g of the total weight of the meal, that of rice 160 g. The GL of the meal consumed with hGI rice (Jasmin Rice) was significantly higher (Table 1). Participants ate less of the medium GI rice on the second test day. However, GL values were not statistically different between both groups (*p* = 0.128). Performing per-protocol analysis, considering only participants who had fully adhered to the study protocol, showed similar results of average serving size (Table S1).

**Table 1:** Gender distribution of the study population and estimated Glycemic Load

	h-mGI (*n* 105)		*p*	m-hGI (*n* 107)		*p*
	Period 1	Period 2		Period 1	Period 2	
Female *n* (%)	47 (44.8)			40 (37.7)		
Glycemic load	99 ± 44	66 ± 33	< 0.001	75 ± 37	100 ± 79	< 0.001

GI, glycemic index; hGI, high GI; mGI, medium GI. Sequence m-hGI: participants received lunch with medium GI rice in the first period and high GI rice in the second period; Sequence h-mGI: vice versa, Paired *t* test, mean ± standard deviation

Not all participants completed all tests: results from two participants were missing for 2-back task and one for alertness. Of the 212 included participants, 33 had to be excluded for task switching analysis due to implausible negative switch costs (at least one of the trials was not finished in time).

### Dietary GI of lunch and cognition

#### Switch task

Intention-to-treat analysis revealed no significant differences between lunches based on mGI or hGI rice for the examined cognitive parameters, switch costs, visual search letters and visual search numbers (Table 2). For two parameters period effects were detected. Switch costs improved in period 2 compared to period 1 in both groups as did the RT for visual search letters. Performing per-protocol analysis showed similar results of cognitive performance (Table S2). None of the tested parameters showed statistically significant differences.

**Table 2:** Cognitive performance in schoolchildren 90 min after eating lunch with medium and high GI rice

	mGI	hGI	Treatment difference (mGI–hGI)	*p*	*p*^d^
Switch (*n* 179)					
Switch costs [s]^b^					
Mean (SE)	167 (4.32)	170 (4.32)	− 2.18 (5.52)	0.693^c^	1.0
95% CI	159, 176	161, 178	− 13.1, 8.71		
Visual search letters [s]^a,b^					
Mean (SE)	10.5 (0.02)	10.5 (0.02)	− 0.004 (0.02)	0.859^c^	1.0
95% CI	10.4, 10.5	10.4, 10.5	− 0.04, 0.04		
Visual search numbers [s]^b^					
Mean (SE)	10.9 (0.02)	10.9 (0.02)	− 0.02 (0.02)	0.218	1.0
95% CI	10.9, 10.9	10.9, 11.0	− 0.05, 0.01		
2-back (*n* 210)					
RT [ms]					
Mean (SE)	463 (9.45)	470 (9.45)	− 6.65 (8.41)	0.430	1.0
95% CI	444, 482	451, 488	− 23.2, 9.93		
Ratio of missings (%)^b^					
Mean (SE)	5.06 (0.14)	5.32 (0.14)	− 0.26 (0.13)	0.047^c^	0.470
95% CI	4.79, 5.33	5.05, 5.59	− 0.51, − 0.003		
Ratio of false alarms (%)^b^					
Mean (SE)	4.38 (0.19)	4.29 (0.19)	0.09 (0.11)	0.388^c^	1.0
95% CI	4.01, 4.75	3.92, 4.66	− 0.12, 0.30		
Alertness (*n* 211)					
Mean RT [ms]^b^					
Mean (SE)	5.67 (0.02)	5.66 (0.02)	0.002 (0.02)	0.886^c^	1.0
95% CI	5.63, 5.70	5.63, 5.70	− 0.03, 0.03		
Deviation of RT [ms]^b^					
Mean (SE)	4.95 (0.04)	4.98 (0.04)	− 0.03 (0.05)	0.557^c^	1.0
95% CI	4.86, 5.03	4.89, 5.06	− 0.12, 0.07		
Count of omission errors (n)^b^					
Mean (SE)	0.81 (0.02)	0.79 (0.02)	0.02 (0.02)	0.365^c^	1.0
95% CI	0.77, 0.84	0.75, 0.83	− 0.02, 0.06		
Count of commission errors (n)^b^					
Mean (SE)	1.55 (0.06)	1.51 (0.06)	0.03 (0.06)	0.540	1.0
95% CI	1.43, 1.67	1.39, 1.63	− 0.08, 0.14		

*CI* confidence interval, *GI* glycemic index, *hGI* high GI, *mGI* medium GI, *RT* reaction time, *SE* standard error of mean

^a^First 12 reactions; Switch costs = (mean RT switch task)-(mean RT number task)-(mean RT 12 reactions of letter task–mean RT first 12 reactions number task)

^b^Transformed with logarithm, square, root, or reciprocal transformation

^c^Period effects detected; analyzed with linear mixed model with fixed effects: GI, sequence, period and random effect: subjects; cognition parameters displayed as predicted values

^d^*p* values Bonferroni–Holm corrected

#### 2-back task

After consumption of lunch with hGI rice the ratio of missings was slightly higher (Table 2). However, after applying Bonferroni correction these differences vanished. The RT and ratio of false alarms did not differ between mGI and hGI. However, the ratio of false alarms improved in period 2 but at the same time ratio of missings increased.

Per-protocol analysis revealed no statistically significant differences in tested parameters between mGI and hGI (Table S2).

#### Alertness

No parameter of the attention task, i.e., RT, deviation of RT, count of omission errors and commission errors differed among children after a lunch of hGI or mGI rice (Table 2). However, period effects were detected. The mean RT and the deviation of RT of the alertness task increased in the second period.

In addition, even after excluding participants who did not follow the protocol, no differences were detected between mGI and hGI.

### Estimated lunch GL

Estimated lunch GL differed significantly between both periods (Table 1). The GL of lunch with hGI rice was higher in both periods compared to that with the GL of lunch with mGI rice. However, models including GL as a covariate revealed no significant GI effects on any cognitive outcome parameter (Table 3). GL was also not significantly associated with these parameters (*p* for all parameters > 0.05). Performing per protocol analysis by excluding participants who had not fully adhered to the study protocol showed no differences of the cognitive performance between both intervention groups as well (Table S3).

**Table 3:** GI effects adjusted for estimated GL on cognitive parameters

	mGI	hGI	Treatment difference (mGI–hGI)	*p*	*p*^d^
Switch (*n* 179)					
Switch costs [s]^b^					
Mean (SE)	167 (4.41)	170 (4.41)	– 2.67 (5.77)	0.644^c^	1.0
95% CI	158, 176	161, 178	– 14.0, 8.71		
Visual search letters [s]^a,b^					
Mean (SE)	10.5 (0.02)	10.5 (0.02)	0.00 (0.02)	0.983^c^	1.0
95% CI	10.4, 10.5	10.4, 10.5	– 0.04, 0.04		
Visual search numbers [s]^b^					
Mean (SE)	10.9 (0.02)	10.9 (0.02)	– 0.01 (0.02)	0.425	1.0
95% CI	10.9, 11.0	10.9, 11.0	– 0.05, 0.02		
2-back (*n* 210)					
RT [ms]					
Mean (SE)	461 (9.62)	471 (9.62)	– 9.88 (9.07)	0.277	1.0
95% CI	442, 480	452, 490	– 27.7, 8.00		
Ratio of missings (%)^b^					
Mean (SE)	5.09 (0.14)	5.29 (0.14)	– 0.21 (0.14)	0.145^c^	1.0
95% CI	4.81, 5.37	5.02, 5.57	– 0.48, 0.71		
Ratio of false alarms (%)^b^					
Mean (SE)	4.41 (0.19)	4.27 (0.19)	0.14 (0.12)	0.234^c^	1.0
95% CI	4.03, 4.78	3.89, 4.64	– 0.09, 0.37		
Alertness (*n* 211)					
Mean RT [ms]^b^					
Mean (SE)	5.67 (0.02)	5.66 (0.02)	0.01 (0.02)	0.458^c^	1.0
95% CI	5.64, 5.71	5.62, 5.70	– 0.02, 0.05		
Deviation of RT [ms]^b^					
Mean (SE)	4.96 (0.04)	4.96 (0.04)	0.002 (0.05)	0.971^c^	1.0
95% CI	4.88, 5.05	4.88, 5.05	– 0.10, 0.10		
Count of omission errors (*n*)^b^					
Mean (SE)	0.80 (0.02)	0.79 (0.02)	0.01 (0.02)	0.587^c^	1.0
95% CI	0.76, 0.84	0.75, 0.83	– 0.03, 0.06		
Count of commission errors (*n*)^b^					
Mean (SE)	1.55 (0.06)	1.51 (0.06)	0.05 (0.06)	0.416	1.0
95% CI	1.43, 1.68	1.38, 1.63	– 0.07, 0.17		

*CI* confidence interval, *GI* glycemic index, *hGI* high GI, *mGI* medium GI, *GL* glycemic load, *RT* reaction time, *SE* standard error of mean

^a^First 12 reactions; Switch costs = (mean RT switch task)-(mean RT number task)-(mean RT 12 reactions of letter task–mean RT first 12 reactions number task)

^b^Transformed with logarithm, square, root, or reciprocal transformation

^c^Period effects detected; analyzed with linear mixed model with fixed effects: GI, GL, sequence, period and random effect: subjects; cognition parameters displayed as predicted values

^d^*p* values Bonferroni–Holm corrected

## Discussion

The current study aimed to investigate whether the dietary GI of lunch had short-term effects on cognitive performance of schoolchildren 90 min after lunch. In our previous CogniDO GI study, we did not see differences in selected cognitive parameters after eating lunch differing in estimated dietary GI at an interval of 45 min within the same crossover approach. Extending the postprandial time to 90 min still had no influence.

Overall, studies on the interrelations of lunch and cognition are scarce, in adults as well as in children. So far, only studies investigating effects of lunch on vigilance per se are available. It was shown that eating lunch can cause a post-lunch dip with impaired cognitive performance in adults [23, 24]. Herein, negative effects of lunch seem to increase with age [25], which we supported in our previous studies showing that children do not suffer from post-lunch dip but even might profit from lunch [13, 18, 19]. The higher glucose metabolism rates of children than of adults may be responsible, because up to the age of about 16 the children’s cerebral cortex requires more glucose compared to adults [26].

However, studies on the estimated dietary GI of lunch and cognition have not been evaluated, yet. The majority of studies investigating GI effects on cognitive performance in children focused on breakfast. For instance, two studies showed that a breakfast with lower GI foods enhanced cognitive functions with respect to reaction speed, accuracy, and in part spatial memory [9, 27]. On the contrary, Smith et al*.* revealed that breakfast with high GI foods improved verbal episodic memory in adolescents (age 14–17 years) after 50 and 90 min [11].

The reasons why our study intervention revealed any cognitive changes are probably manifold. The main difference between studies on breakfast and lunch is that influences of foods with different GI consumed at breakfast were examined after overnight fasting. The glycemic response after a fasting period for 8–12 h might be more pronounced. For instance, Ogata et al*.* showed that skipping breakfast leads to much more pronounced glycemic response after eating lunch in healthy young individuals [28], thus fasting time before having a meal seems to be of relevance. This is in line with a recent study of 10–13-year-old children in a school-based testing environment, which showed that an ad libitum breakfast improved reaction speed, visual-sustained attention, and visual–spatial memory [29]. We decided to serve a breakfast approximately 3 h before lunch to ensure that all children participate under almost the same conditions and avoid differences of fasting times, because many children (approximately 13–25%) in Germany skip breakfast [30, 31]. Typically, blood glucose concentrations return to baseline or below 3–4 h after ingestion. Thus, the time frame between breakfast and lunch might have been too short in our intervention design for blood glucose levels to return to baseline.

Overall, it is difficult to draw conclusions about the acute effects of lunch composition on cognition. The conflicting results of breakfast studies with children on attention and working memory tasks suggest that metabolic criteria such as body weight and the glucose-mediated insulin response, as well as intervention adherence and lunch type are relevant [15, 32]. Another important factor is the GI difference between both rice types. Based on data from others, there can be significant differences in the GI between basmati rice and jasmine rice (low GI vs. high GI) [33]. These rice types have already been chosen for our pervious study after testing for sensory properties and their acceptance by children. To ensure comparability with the current study the same rice types were used. However, due to climatic influences or different growing areas, the starch content can differ from year to year potentially affecting the GI [34] so that the rice GI’s were determined again for the present study. GI-values changed from formerly 62 vs. 86 (Basmati vs. Jasmine rice) to 64 vs. 79. Possibly, the differences between both rice types with regard to the GI were not sufficient to detect effects on cognition.

### Strengths and limitations

A strength of this study is that children’s cognitive performance was tested under real life conditions within their everyday school environment and by choosing a habitual dish with roughly equal macronutrient composition in the two foods with different GI, instead of artificial food preparations. A controlled laboratory environment might have delivered more unequivocal results, but would have lacked transferability into everyday life. Nevertheless, we chose to analyze the selected rice types in a certified lab according to ISO standards instead of solely relying on theoretical reference values.

However, the study has also some limitations. Although breakfast was standardized in terms of food components, the amount of breakfast consumed was not controlled thereby potentially influencing the outcome. Equally important, blood glucose regulation varies individually [15]. Baseline and post-intervention glucose measurements as well as information on body composition (e.g., body mass index) would have been useful to better understand the relationship between GI, blood glucose and cognition [6]. However, it is a common difficulty in pediatrics that parents are not willing to consent to extensive examination, particularly in the case of healthy children. Furthermore, our study design lacks baseline and late postprandial cognitive assessments. However, multiple testing might have caused even more learning effects and the children’s motivation would have probably decreased. In addition, the cognitive test run lasted 45 min so that cognition was recorded in a time frame of 90–135 min. Together with our previous study in which we examined the same cognitive parameters 45 min after lunch [12], a very broad time window was covered.

## Conclusion

In conclusion, the present study supports the hypothesis that the dietary GI of carbohydrate-rich foods consumed at lunch has no effects on children’s cognitive performance after 90 min in a school setting. Short-term postprandial effects of the GI of foods on cognition might be of more relevance in the morning. Whether other nutrients have short-term effects on cognition in children under real-life conditions, needs to be established. In addition, other physiological and anatomical characteristics of the participants should be investigated in this context.

## Supplementary Information

Below is the link to the electronic supplementary material.

Supplementary file1 (DOCX 24 KB)
