# Supplementary material for: Impact of lunch with carbohydrates differing in glycemic index on children's cognitive functioning in the late postprandial phase: a randomized crossover study
Source: Eur J Nutr. 2021 Dec 13;61(3):1637–47. doi: 10.1007/s00394-021-02766-y (PMC8921027; doi:10.1007/s00394-021-02766-y)
Supplement: Supplementary file 1 — Supplementary file1 (DOCX 24 KB) [file 394_2021_2766_MOESM1_ESM.docx]

**Table S1** Gender distribution of the study population and estimated Glycemic Load

|  | **h-mGI (*n* 94)** | |  | **m-hGI (*n* 96)** | |  |
| --- | --- | --- | --- | --- | --- | --- |
|  | **Period 1** | **Period 2** | ***p*** | **Period 1** | **Period 2** | ***p*** |
| **Female *n* (%)** | 41 (43.6) | |  | 37 (38.5) | |  |
| **Glycemic Load** | 99 ± 44 | 66 ± 33 | < 0.001 | 75 ± 37 | 98 ± 82 | < 0.001 |

GI, glycemic index; hGI, high GI; mGI, medium GI. Sequence m-hGI: participants received lunch with medium GI rice in the first period and high GI rice in the second period; Sequence h-mGI: *vice versa*, Paired *t* test, mean ± standard deviation.

**Table S2** Per protocol analysis of cognitive performance in schoolchildren 90 minutes after eating lunch with medium and high GI rice

|  | **mGI** | **hGI** | **Treatment difference**  **(mGI-hGI)** | ***p*** | ***p*^d^** |
| --- | --- | --- | --- | --- | --- |
| **Switch (*n* 160)** |  |  |  |  |  |
| **Switch costs [s]^b^** |  |  |  |  |  |
| Mean (SE) | 169 (4.65) | 168 (4.65) | 0.35 (5.75) | 0.951^c^ | 1.0 |
| 95% CI | 160, 178 | 159, 177 | -11.0, 11.7 |  |  |
| **Visual search letters [s]^a,b^** |  |  |  |  |  |
| Mean (SE) | 10.5 (0.02) | 10.5 (0.02) | -0.02 (0.02) | 0.267^c^ | 1.0 |
| 95% CI | 10.4, 10.5 | 10.5, 10.5 | -0.06, 0.02 |  |  |
| **Visual search numbers [s]^b^** |  |  |  |  |  |
| Mean (SE) | 10.9 (0.02) | 10.9 (0.02) | -0.02 (0.02) | 0.191 | 1.0 |
| 95% CI | 10.9, 11.0 | 10.9, 11.0 | -0.05, 0.01 |  |  |
| **2-back (*n* 189)** |  |  |  |  |  |
| **RT [ms]** |  |  |  |  |  |
| Mean (SE) | 465 (9.85) | 468 (9.85) | -3.56 (8.82) | 0.687 | 1.0 |
| 95% CI | 445, 484 | 449, 487 | -21.0, 13.8 |  |  |
| **Ratio of missings (%)^b^** |  |  |  |  |  |
| Mean (SE) | 5.07 (0.15) | 5.31 (0.15) | -0.25 (0.14) | 0.070^c^ | 0.700 |
| 95% CI | 4.78, 5.35 | 5.02, 5.06 | -0.52, 0.02 |  |  |
| **Ratio of false alarms (%)^b^** |  |  |  |  |  |
| Mean (SE) | 4.39 (0.19) | 4.30 (0.19) | 0.10 (0.12) | 0.381^c^ | 1.0 |
| 95% CI | 4.01, 4.78 | 3.92, 4.68 | -0.12, 0.31 |  |  |
| **Alertness (*n* 190)** |  |  |  |  |  |
| **Mean RT [ms]^b^** |  |  |  |  |  |
| Mean (SE) | 5.67 (0.02) | 5.67 (0.02) | -0.01 (0.02) | 0.740^c^ | 1.0 |
| 95% CI | 5.63, 5.70 | 5.63, 5.71 | -0.04, 0.03 |  |  |
| **Deviation of RT [ms]^b^** |  |  |  |  |  |
| Mean (SE) | 4.95 (0.05) | 5.00 (0.05) | -0.05 (0.05) | 0.344^c^ | 1.0 |
| 95% CI | 4.86, 5.04 | 4.91, 5.09 | -0.15, 0.05 |  |  |
| **Count of omission errors (n)^b^** |  |  |  |  |  |
| Mean (SE) | 0.80 (0.02) | 0.79 (0.02) | 0.02 (0.02) | 0.444^c^ | 1.0 |
| 95% CI | 0.76, 0.85 | 0.75, 0.83 | -0.03, 0.06 |  |  |
| **Count of commission errors (n)^b^** |  |  |  |  |  |
| Mean (SE) | 1.55 (0.07) | 1.55 (0.07) | -0.01 (0.06) | 0.934 | 1.0 |
| 95% CI | 1.42, 1.67 | 1.42, 1.68 | -0.12, 0.11 |  |  |

CI, confidence interval; GI, glycemic index; hGI, high GI; mGI, medium GI; RT, reaction time; SE, standard error of mean; ^a^ First twelve reactions; Switch costs = (mean RT switch task)-(mean RT number task)-(mean RT twelve reactions of letter task – mean RT first twelve reactions number task); ^b^ transformed with logarithm, square, root, or reciprocal transformation; ^c^ period effects detected; analyzed with linear mixed model with fixed effects: GI, sequence, period and random effect: subjects; cognition parameters displayed as predicted values; ^d^ *p*-values Bonferroni-Holm corrected.

**Table S3** Per protocol analysis of GI effects adjusted for estimated GL on cognitive parameters

CI, confidence interval; GI, glycemic index; hGI, high GI; mGI, medium GI; GL, glycemic load; RT, reaction time; SE, standard error of mean; ^a^ First twelve reactions; Switch costs = (mean RT switch task)-(mean RT number task)-(mean RT twelve reactions of letter task – mean RT first twelve reactions number task); ^b^ transformed with logarithm, square, root, or reciprocal transformation; ^c^ period effects detected; analyzed with linear mixed model with fixed effects: GI, GL, sequence, period and random effect: subjects; cognition parameters displayed as predicted values; ^d^ p-values Bonferroni-Holm corrected.

|  | **mGI** | **hGI** | **Treatment difference**  **(mGI-hGI)** | ***p*** | ***p*^d^** |
| --- | --- | --- | --- | --- | --- |
| **Switch (*n* 160)** |  |  |  |  |  |
| **Switch costs [s]^b^** |  |  |  |  |  |
| Mean (SE) | 168 (4.74) | 169 (4.73) | -0.06 (6.01) | 0.993^c^ | 1.0 |
| 95% CI | 159, 178 | 159, 178 | -11.9, 11.8 |  |  |
| **Visual search letters [s]^a,b^** |  |  |  |  |  |
| Mean (SE) | 10.5 (0.02) | 10.5 (0.02) | -0.02 (0.02) | 0.370^c^ | 1.0 |
| 95% CI | 10.4, 10.5 | 10.4, 10.6 | -0.06, 0.02 |  |  |
| **Visual search numbers [s]^b^** |  |  |  |  |  |
| Mean (SE) | 10.9 (0.02) | 10.9 (0.02) | -0.02 (0.02) | 0.368 | 1.0 |
| 95% CI | 10.9, 11.0 | 10.9, 11.0 | -0.05, 0.02 |  |  |
| **2-back (*n* 189)** |  |  |  |  |  |
| **RT [ms]** |  |  |  |  |  |
| Mean (SE) | 463 (10.0) | 470 (10.0) | -6.63 (9.46) | 0.484 | 1.0 |
| 95% CI | 443, 483 | 450, 489 | -25.3, 12.0 |  |  |
| **Ratio of missings (%)^b^** |  |  |  |  |  |
| Mean (SE) | 5.09 (0.15) | 5.29 (0.15) | -0.20 (0.15) | 0.167^c^ | 1.0 |
| 95% CI | 4.79, 5.38 | 5.00, 5.58 | -0.49, 0.09 |  |  |
| **Ratio of false alarms (%)^b^** |  |  |  |  |  |
| Mean (SE) | 4.42 (0.20) | 4.27 (0.20) | 0.15 (0.12) | 0.202^c^ | 1.0 |
| 95% CI | 4.04, 4.81 | 3.88, 4.66 | -0.08, 0.39 |  |  |
| **Alertness (*n* 190)** |  |  |  |  |  |
| **Mean RT [ms]^b^** |  |  |  |  |  |
| Mean (SE) | 5.67 (0.02) | 5.67 (0.02) | 0.01 (0.02) | 0.775^c^ | 1.0 |
| 95% CI | 5.63, 5.71 | 5.63, 5.70 | -0.03, 0.04 |  |  |
| **Deviation of RT [ms]^b^** |  |  |  |  |  |
| Mean (SE) | 4.96 (0.05) | 4.98 (0.05) | -0.02 (0.05) | 0.755^c^ | 1.0 |
| 95% CI | 4.87, 5.06 | 4.89, 5.07 | -0.12, 0.09 |  |  |
| **Count of omission errors (n)^b^** |  |  |  |  |  |
| Mean (SE) | 0.80 (0.02) | 0.79 (0.02) | 0.01 (0.02) | 0.676^c^ | 1.0 |
| 95% CI | 0.76, 0.84 | 0.75, 0.83 | -0.04, 0.06 |  |  |
| **Count of commission errors (n)^b^** |  |  |  |  |  |
| Mean (SE) | 1.55 (0.07) | 1.54 (0.07) | 0.01 (0.06) | 0.857 | 1.0 |
| 95% CI | 1.42, 1.68 | 1.41, 1.67 | -0.11, 0.13 |  |  |
